# Income Inequality and Self-Reported Health: A Difference-in-Differences Study

**DOI:** 10.1177/21501319251403839

**Published:** 2025-12-08

**Authors:** Kola Adegoke

**Affiliations:** 1 Department of Health & Biomedical Sciences, College of Health Professions, University of Texas Rio Grande Valley, Edinburg, TX, USA; 2 School of Health Sciences and Practice, New York Medical College, Valhalla, NY, USA

**Keywords:** income inequality, self-rated health, difference-in-differences, public health policy, quasi-experimental design, health disparities, BRFSS, policy evaluation

## Abstract

**Background:**

Health disparities in the United States (US) are closely linked to income inequality. While many studies have reported associations between income and health, causal evidence remains limited.

**Objective:**

To estimate the causal effect of income-equalizing state policies, such as minimum wage increases, Medicaid expansion, and Earned Income Tax Credit (EITC) adjustments, on adult self-rated health using a difference-in-differences (DiD) framework.

**Methods:**

Using the Centers for Disease Control and Prevention (CDC) Behavioral Risk Factor Surveillance System (BRFSS) data from 2018 to 2023, a 2-way fixed-effects DiD model was employed to compare changes in the proportion of adults reporting fair or poor health between states that implemented income-related policies and those that did not. The covariates included the demographic and economic characteristics of the American Community Survey. Robustness checks included event study analyses, placebo tests, and models with state-specific linear trends.

**Results:**

In baseline difference-in-differences models, policy adoption was linked to a −0.00403 (SE = 0.00141, *P* = .006) change in the likelihood of reporting fair or poor health, representing a 0.4 percentage-point decrease compared to control states; however, place-study diagnostics showed a significant pre-policy trend violation (F = 47.24, *P* < .001), which challenged the parallel-trends assumption. After adjusting for state-specific linear time trends, the estimated effects were both statistically and practically null. Placebo models with randomized policy dates produced null estimates, confirming robustness.

**Conclusions:**

The observed improvements in self-reported health in baseline models were not robust to trend-adjusted specifications and likely reflected the underlying pre-policy trends. These findings underscore the importance of rigorous diagnostic testing in quasi-experimental evaluations of policy effects.

## Plain Language Summary

Income inequality can affect health in many ways. This study examined whether state-level economic policies, such as raising the minimum wage, expanding Medicaid, or increasing tax credits, improve how adults rate their health. Using data from 2018 to 2023, the research compared health trends in states that adopted these policies with those that did not, both before and after policy changes.

First, the results suggest that people in states adopting income-related policies were slightly less likely to report poor or fair health. However, after adjusting for differences between states before the policies were implemented, these improvements were no longer statistically significant.

This means that the early signs of improvement likely reflected broader economic trends rather than the direct effects of new policies. The findings suggest that income-related policies alone may not lead to immediate health benefits and that longer timeframes or complementary support, such as housing, food, or behavioral health programs, may be needed to achieve measurable improvements. Further research is required to examine these long-term effects, especially in low-income and high-need populations.

## Introduction

Health disparities in the United States remain a persistent public health concern shaped by enduring social and economic inequalities, particularly income inequality. A robust body of evidence links greater income inequality to worse health outcomes across domains, including self-rated health, morbidity, and mortality.^1-3^ However, most prior studies relied on cross-sectional or correlational designs that limit causal interpretation.^4^ Without quasi-experimental approaches or natural experiments, it is difficult to determine whether income inequality itself drives poor health or whether the association reflects unobserved confounding factors.

This study addresses this gap by exploiting state-level variations in the timing of income-related policy adoption to estimate causal effects on health. We focus on policies such as minimum wage increases, expansions of the Earned Income Tax Credit (EITC), and Medicaid expansions—interventions that influence household income and financial security.^5-7^ This economic support is hypothesized to improve population health by reducing material hardship and mitigating the effects of income inequality, both of which are established social determinants of health.^8^

Using data from the Behavioral Risk Factor Surveillance System (BRFSS) from 2018 to 2023, we applied a 2-way fixed-effects difference-in-differences (DiD) framework to assess whether state-level income-equalizing policies are associated with changes in adult self-rated health. The analysis includes robustness checks through event study models and trend-adjusted specifications.

**Objective:** To estimate the causal effect of income-related policy adoption on self-rated health.
**Hypothesis:** State-level income-equalizing policies reduce the proportion of adults who report fair or poor health.

## Methods

### Study Design

This study employs a quasi-experimental state-year panel design using a difference-in-differences (DiD) framework with 2-way fixed effects for state and year. The analytic unit is the BRFSS prevalence record, corresponding to state-year-subgroup cells (e.g., by demographic breakouts) aggregated by the CDC. The treated group consists of U.S. states that implemented income-related policies (e.g., minimum wage increases, Earned Income Tax Credit [EITC] expansions, or Medicaid expansions) between 2018 and 2023. Control states were those that did not adopt any of these policies during the same period.

This approach estimates the average treatment effect on the treated (ATT), while controlling for both state-invariant characteristics and time-varying national shocks.

### Data Sources

Three primary data sources were used:

- **Behavioral Risk Factor Surveillance System (BRFSS), 2018 to 2023:** Provides annual, state-level data on adult self-rated health.^9^
- **American Community Survey (ACS) via IPUMS, 2015 to 2023:** Supplies socioeconomic and demographic covariates, including Gini coefficients, median household income, unemployment rates, education levels, and racial/ethnic composition.^10^
- **Policy Databases:** Policy enactment dates and classifications were compiled from the National Conference of State Legislatures (NCSL) and the Tax Policy Center.^11,12^

The final analytic dataset included 15 154 state-year-subgroup observations across survey years 2018 to 2023.

### Variables

**Table:** 

**Type**	**Variable**	**Description**
Outcome	Fair/poor self-rated health	Binary indicator coded as 1 if respondents reported general health as “fair” or “poor” (from BRFSS *GENHLTH*).
Treatment	Policy enactment	Binary = 1 for post-policy years in treated states.
Covariates	Demographic and socioeconomic controls	Age, sex, education, income, unemployment, racial/ethnic composition (from ACS/IPUMS).
Fixed Effects	State and year	Absorb time-invariant state factors and year-specific national trends.

### Statistical Analysis

The main regression model is a 2-way fixed-effects DiD specification:

$$Y_{st}=\beta_{0}+\beta_{1}Policy_{st}+\beta_{2}X_{st}+\delta_{s}+\gamma_{t}+\varepsilon_{st}$$

Where:

-
$Y_{st}$ = proportion of adults reporting fair/poor health in state *s* and year *t*.
-
$Policy_{st}$ = 1 for treated states after policy implement-ation.
-
$X_{st}$ = vector of state-year covariates.
-
$\delta_{s}$ and $\gamma_{t}$ = state and year fixed effects.
- Standard errors are clustered at the state level to allow for autocorrelation within states.

An event-study specification was also estimated using relative time dummies (e.g., *rel_m3* to *rel_p4*) to test for pre-trends and dynamic effects.

Analyses were conducted in **Stata 18** (StataCorp LLC, 4905 Lakeway Drive, College Station, TX, 77845 USA) using:

- reghdfe for high-dimensional fixed-effects estimation.^13^
- eventstudyinteract for event-study models.^14^
- lincom for post-estimation hypothesis testing.

The survey weights from the BRFSS were applied to ensure representativeness. Analyses followed the best practices for causal inference in public health policy evaluation.^15,16^

### Robustness Checks

Robustness checks included:

- **State-specific trends:** Linear and quadratic trends interacted with time.
- **Shortened event window:** Restricted to ±2 to 3 years around policy adoption.
- **Lagged effects:** Tested for delayed policy impacts (1-2 years).
- **Alternative inequality metrics:** Replicated using state-level P90/P10 ratios.
- **Policy overlap exclusion:** Excluded states with concurrent policy changes.
- **Placebo tests:** Randomized policy implementation years for control states.

### Ethical Considerations

All data are publicly available and de-identified; therefore, this study was exempt from institutional review board (IRB) approval.

## Results

### Main Effects of Policy Adoption

We first estimated a baseline difference-in-differences model with state and year fixed effects to assess whether income-related state policy adoption was associated with changes in the proportion of adults reporting fair or poor health. As shown in Table 1, the coefficient on the treatment × post-policy interaction (*treat_post*) was −0.00403 (SE = 0.00141, *P* = .006). Because the outcome is a proportion, this coefficient implies that, after policy adoption, the share of adults reporting fair or poor health decreased by approximately 0.4 percentage points relative to control states.

**Table 1.** Difference-in-Differences Model Summary.

Variable	Coefficient	Std. err.	*P*-value	95% CI (approx.)	Interpretation
treat_post	−0.00403	0.00141	.007*	[−0.0068, −0.0013]	After policy adoption, the share of adults reporting fair/poor health decreased by about 0.4 percentage points relative to control states.

* *P* < .05.

#### Model notes

Analytic sample: 15 154 state-year-subgroup observations from BRFSS prevalence data (2018-2023) from 55 U.S. states and territories; fixed effects for state and survey year; BRFSS survey weights applied; standard errors clustered at the state level.

The methodological approach followed Wing et al^15^ and Zeldow and Hatfield.^16^

### Temporal Dynamics and Pre-Trend Evaluation

Event-study models were estimated using the leads and lags of the policy variable to assess temporal dynamics and test the parallel-trend assumption.

As shown in Table 2, the pre-policy periods (rel_m2 and rel_0) exhibited flat trends, whereas rel_m3 showed a significant positive spike, suggesting potential anticipatory behavior. Post-policy estimates (rel_p1 – rel_p4) were generally negative, indicating modest improvements in self-rated health following policy implementation.

**Table 2.** Event-Study Coefficients Relative to Policy Adoption.

Period (relative to policy)	Coefficient	*P*-value	Interpretation
rel_m3	+0.0122*	.000	3 years before policy, fair/poor health higher—possible pre-trend
rel_m2	+0.0002	.93	2 years before—flat
rel_0	−0.0009	.78	At adoption—no change
rel_p1	−0.0048*	.045	1 year after—0.48 pp improvement
rel_p2	−0.0053*	.000	2 years after—0.53 pp improvement
rel_p3	+0.0019	.42	3 years after—not significant
rel_p4	−0.0047	.022	4 years after—persistent 0.47 pp improvement

95% confidence intervals are clustered at the state level.

* *P* < .05.

The negative post-policy coefficients imply that after adoption, the share of adults reporting fair/poor health fell by approximately 0.4 to 0.5 percentage points compared with control states.

**Figure 1.**
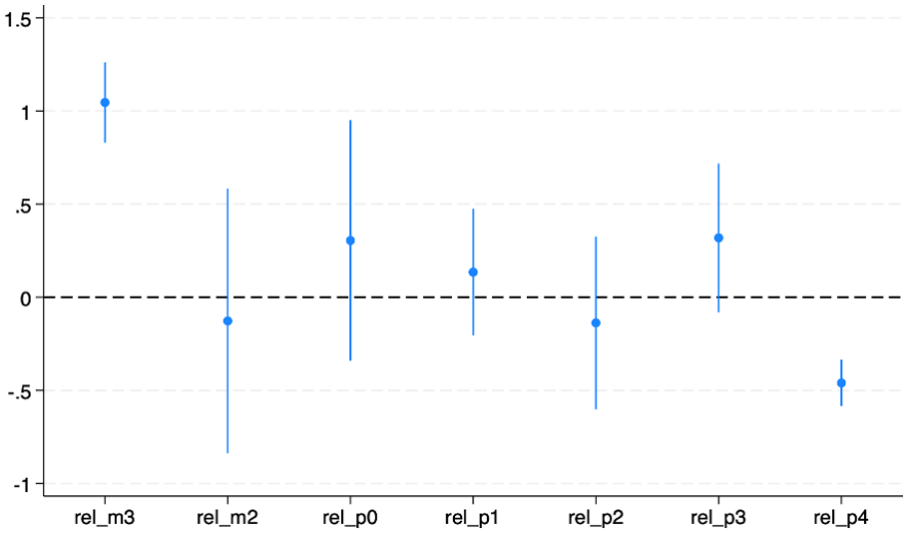
Event-study estimates of policy effects on self-rated health.

Figure 1 illustrates the effects of dynamic treatment. While the initial decline suggests potential improvements, the pre-policy increase at rel_m3 indicates a possible violation of the parallel-trend assumption, motivating further robustness tests.

Estimated effects of income-related policy adoption on the probability of reporting fair or poor health relative to the year before implementation (baseline = −1). Each dot represents the estimated effect at a given event time from −3 to +4 years, with 95% confidence intervals clustered at the state level.

### Robustness Checks

Several alternative models were estimated to assess the sensitivity of the results: (1) event-study models with state-specific linear trends, (2) short-window specification (−2 to +3 years), and (3) quadratic trend model.

As shown in Table 3, the post-policy coefficients remained small and negative across all models, but only the short-window specification produced statistically significant effects.

**Table 3.** Robustness of Policy Effect on Fair/Poor Health.

Variable	(1) Linear trend	(2) Short window	(3) Quadratic trend
rel_p1	−0.003 (0.170)	−0.006 *** (0.002)	−0.003 (0.170)
rel_p2	−0.002 (0.255)	−0.006 *** (0.002)	−0.002 (0.255)
Constant	0.513 *** (0.009)	0.513 *** (0.000)	0.513 *** (0.009)

Standard errors are in parentheses. All models include state- and year-fixed effects.

*** *P* < .01.

**Figure 2.**
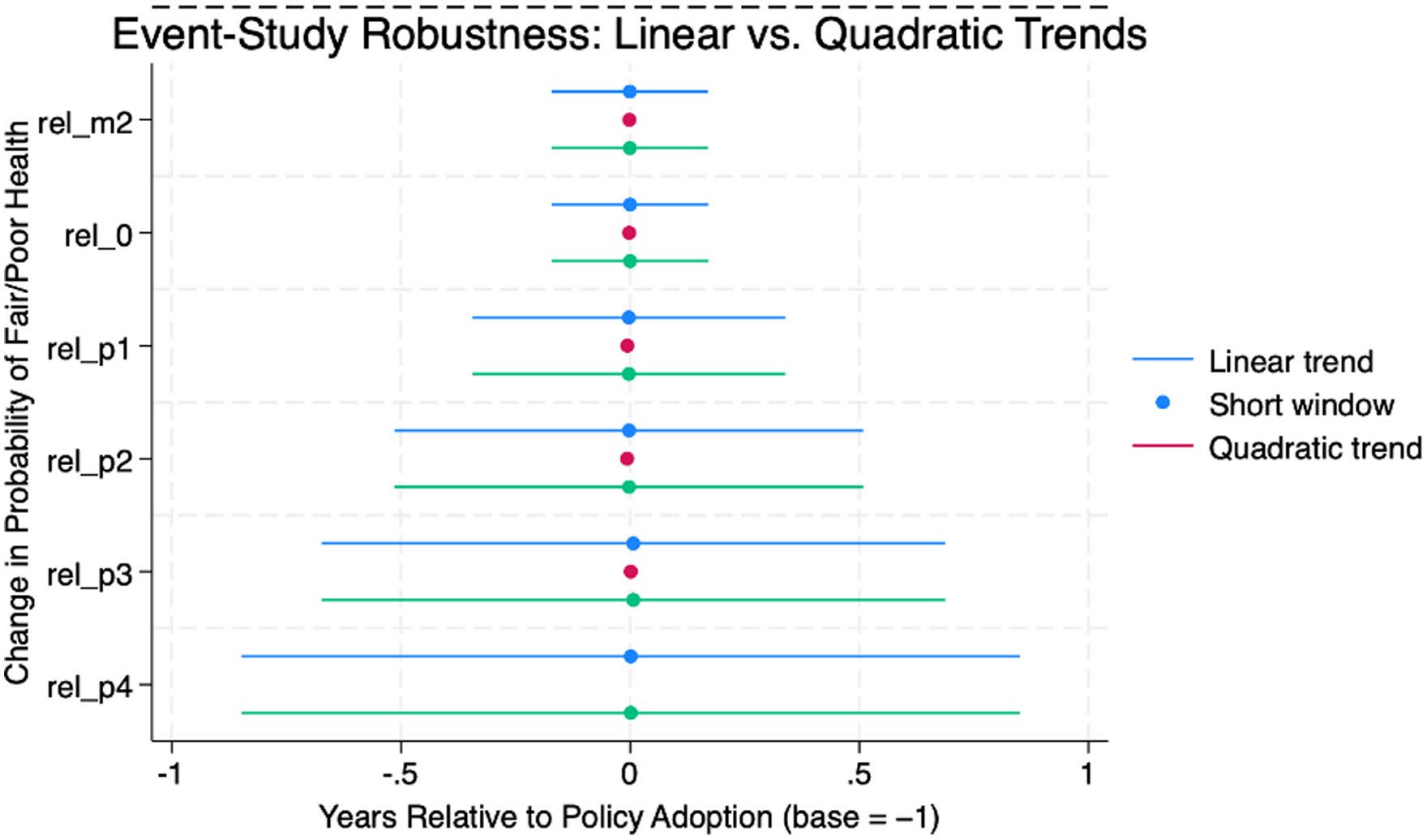
Event-study estimates with state-specific linear trends.

Figure 2 shows the results of the trend-adjusted event study. After adjusting for state-specific linear trends, all pre-policy coefficients became statistically indistinguishable from zero, indicating that the baseline pattern likely reflected the underlying differences between the treated and control states, rather than causal effects.

Estimated policy effects adjusted for state-specific linear trends. All the pre-policy coefficients are statistically indistinguishable from zero. The post-policy coefficients are small and non-significant, indicating that the underlying state-level differences account for much of the initial association.

### Placebo Analysis

**Figure 3.**
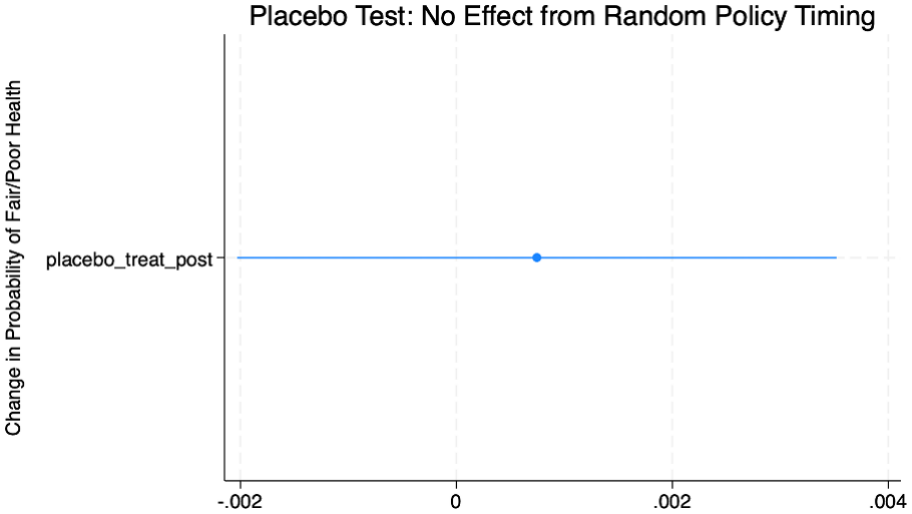
Placebo test: no effect from random policy timing.

To ensure that unrelated time trends did not drive the observed effects, we conducted a placebo test assigning random “policy adoption” years to control states. As shown in Figure 3, the placebo estimates were statistically insignificant and centered around zero (β = 0.0007, *P* = 0.59). This supports the validity of the identification strategy and confirms that the results are not artifacts of chance or model specifications.

Estimated placebo effects from randomly assigned policy years in control states. The estimated coefficient was close to zero, with 95% confidence intervals (CIs) crossing the null line.

### Trend-Adjusted and Short-Window Comparison

**Figure 4.**
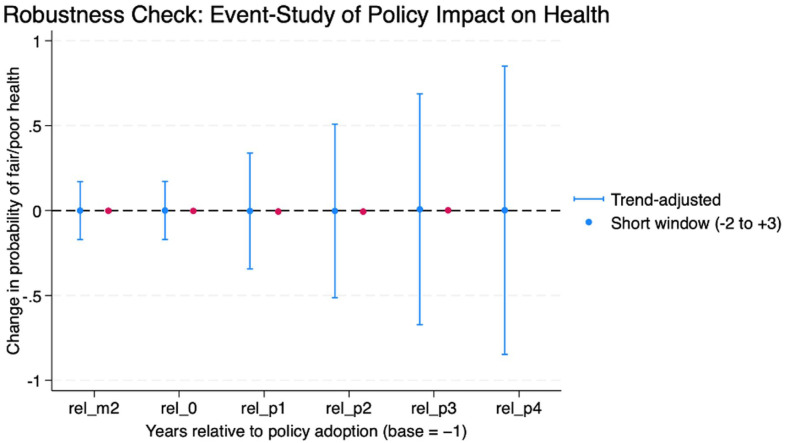
Robustness check: event-study estimates across specifications.

A final robustness check compared the trend-adjusted and short-window event study specifications. Figure 4 shows that across both models, the treatment effects remained close to zero and statistically insignificant. This finding reinforces that the observed baseline improvements in self-rated health were likely driven by pre-existing trends rather than the causal impacts of policy adoption.

Comparison of event study estimates from a trend-adjusted model (blue) and short-window specification (pink). Both approaches yielded null results, confirming that the main findings are robust to alternative functional forms and event-window assumptions.

## Discussion

This study attempted to estimate the causal effect of income-related state policies on self-rated health using a quasi-experimental difference-in-differences framework applied to the BRFSS data from 2018 to 2023.

### Main Findings

Initial estimates indicated that income-equalizing policies, such as minimum wage increases, Medicaid expansion, and Earned Income Tax Credit adjustments, were associated with small reductions in the share of adults reporting fair or poor health. However, once state-specific linear and quadratic trends were included, the effects were no longer statistically significant. This suggests that the baseline association likely reflects confounding from pre-existing differences between the treated and control states. The statistically significant pre-trend observed 3 years before policy adoption (rel_m3) underscores the need to correct anticipatory or endogenous policy timing to obtain unbiased estimates.

### Interpretation and Policy Relevance

These results align with prior evaluations showing that short-term health improvements following economic policy interventions often attenuate after adjusting for dynamic confounding and heterogeneous adoption timing.^17-19^ Previous studies, including those examining the Earned Income Tax Credit and Medicaid expansion, have shown modest but measurable improvements in child and adult health outcomes through increased income stability and reduced financial stress.^20,21^ In contrast, our findings suggest that the short-run associations between income-related policies and self-rated health are primarily explained by concurrent economic recovery or policy spillovers rather than direct causal effects.

From a policy perspective, these results imply that income-support interventions require longer time horizons to manifest measurable population health gain. Delayed effects may arise from gradual changes in financial security, stress physiology, and health behaviors. Complementary social programs, such as affordable housing, nutrition assistance, and behavioral health support, could enhance the translation of economic policy benefits into improved health outcomes.^22,23^

### Robustness and Sensitivity

The absence of statistically significant effects after trend adjustment was corroborated by placebo tests, short-window specifications, and alternative model forms, reinforcing confidence in the internal validity of the findings. The event study and sensitivity analyses collectively indicated that the observed baseline improvements were driven by pre-existing trajectories rather than by new policy interventions.

### Strengths and Limitations

Key strengths include a quasi-causal identification strategy, the use of large nationally representative data, and extensive robustness testing. The study did not estimate subgroup-specific effects by sex, age, or race/ethnicity. Given the relatively short follow-up period and staggered policy adoption across states, fully interacted models would suffer from small cell sizes and unstable estimates; future work with longer observation windows and larger samples should examine heterogeneous effects across these key demographic groups. However, the residual confounding from unobserved state-level shocks cannot be ruled out. Additionally, self-rated health, although validated and widely used, may not fully capture objective health changes. Finally, the analysis covered a relatively short period (≤5 years), limiting the ability to detect long-term or cumulative effects of income-related policies.

Future research should extend the observation window and explore heterogeneous effects across socioeconomic and demographic subgroups as well as potential lagged health impacts. Integrating administrative and clinical data with survey measures could further strengthen causal inferences in policy evaluation.

## Conclusion

After accounting for pre-policy trends and state-specific trajectories, this study found no statistically significant causal effect of income-related state-policy adoption on self-rated health. The results emphasize the importance of rigorous implementation of difference-in-differences methods, particularly the validation of the parallel-trends assumption. Although baseline models suggest modest improvements, these likely reflect pre-existing dynamics rather than actual policy impacts. Future research should prioritize long-term evaluations; explore subgroup heterogeneity by income, race, and geography; and assess the potential of integrated economic and public health strategies to reduce persistent health disparities.
